# Limited Diagnostic Utility of Chromogranin A Measurements in Workup of Neuroendocrine Tumors

**DOI:** 10.3390/diagnostics10110881

**Published:** 2020-10-29

**Authors:** Jonas Baekdal, Jesper Krogh, Marianne Klose, Pernille Holmager, Seppo W. Langer, Peter Oturai, Andreas Kjaer, Birgitte Federspiel, Linda Hilsted, Jens F. Rehfeld, Ulrich Knigge, Mikkel Andreassen

**Affiliations:** 1 ENETS Neuroendocrine Tumor Centre of Excellence, Rigshospitalet, Copenhagen University Hospital, 2100 Copenhagen, Denmark; jesper.krogh@dadlnet.dk (J.K.); marianne.christina.klose.01@regionh.dk (M.K.); pernille.holmager.01@regionh.dk (P.H.); Seppo.Langer@regionh.dk (S.W.L.); Peter.Sandor.Oturai@regionh.dk (P.O.); akjaer@sund.ku.dk (A.K.); birgitte.federspiel@regionh.dk (B.F.); linda.maria.hilsted@regionh.dk (L.H.); Jens.F.Rehfeld@regionh.dk (J.F.R.); Ulrich.Peter.Knigge@regionh.dk (U.K.); mikkel.andreassen.01@regionh.dk (M.A.); 2 Department of Endocrinology, Rigshospitalet, Copenhagen University Hospital, 2100 Copenhagen, Denmark; 3 Department of Oncology, Rigshospitalet, Copenhagen University Hospital, 2100 Copenhagen, Denmark; 4 Department of Clinical Physiology, Nuclear Medicine & PET and Cluster for Molecular Imaging, Copenhagen University Hospital, 2100 Copenhagen, Denmark; 5 Department of Biomedical Sciences, Rigshospitalet and University of Copenhagen, 2100 Copenhagen, Denmark; 6 Department of Pathology, Rigshospitalet, Copenhagen University Hospital, 2100 Copenhagen, Denmark; 7 Department of Clinical Biochemistry, Rigshospitalet, Copenhagen University Hospital, 2100 Copenhagen, Denmark; 8 Department of Surgery and Transplantation, Rigshospitalet, Copenhagen University Hospital, 2100 Copenhagen, Denmark

**Keywords:** chromogranin A, neuroendocrine tumor, workup, processing-independent analysis (PIA), positive predictive value (PPV)

## Abstract

Background: Plasma chromogranin A (CgA) is related to tumor burden and recommended in the follow-up of patients diagnosed with neuroendocrine tumors (NETs). The use of CgA in the workup of a suspected NET is more questionable. Objective: To assess the positive predictive value (PPV) of CgA plasma concentrations above the upper reference limit (URL) in patients with suspected NET. Method: Patients referred to the NET Centre, Rigshospitalet, Copenhagen from 2015 to 2019 with clinically suspected NET were included if a CgA measurement was performed prior to referral. The utility of CgA was assessed by comparing pre-referral CgA concentrations to the outcome of a thorough workup. In 47 selected cases with continuously unexplained elevated CgA concentrations, a processing-independent analysis (PIA) for CgA was performed. Results: A total of 197 patients were included. NET was ultimately diagnosed in 25 patients. CgA plasma concentrations were above the URL (elevated) in 19/25 patients diagnosed with NET. In total, 167/197 had elevated CgA concentrations at referral. The positive predictive value (PPV) of elevated CgA concentration was 11% (19/167). Proton pump inhibitor (PPI) treatment was identified as the possible cause of CgA elevation in 55/148 patients with falsely elevated CgA. CgA concentration was normal in 28/47 patients when using PIA. Conclusion: Our data do not support using measurement of CgA for screening when NET is suspected since the PPV was rather low. PPI treatment is a common cause of increased CgA concentrations and should always be discontinued before CgA measurement. PIA of CgA could be a way of excluding NET when suspicion is based primarily on elevated CgA.

## 1. Introduction

Neuroendocrine neoplasms (NENs) are rare heterogenous tumors which may arise in several different anatomical sites such as small intestine, pancreas and lungs [1]. Well-differentiated tumors are referred to as neuroendocrine tumors (NETs) whereas poorly differentiated NENs are referred to as neuroendocrine carcinomas (NECs). NETs are further divided, according to Ki-67 proliferation index, into low- (G1) (Ki-67 < 3%), intermediate- (G2) (Ki-67 3–20%) or high-grade (G3) (Ki-67 > 20%) [2]. NENs arise from neuroendocrine cells which express markers of neuroendocrine differentiation (e.g., chromogranin A (CgA) and synaptophysin) [3]. Some of these tumors, particularly G1 and G2 NETs, are functional—i.e., able to produce and release bioactive peptides or amines to the bloodstream in contrast to non-functional NETs [4,5]. The release of bioactive peptide and amines causes specific endocrine symptoms related to the specific molecule (e.g., hypergastrinemia due to a gastrinoma), which may lead to an earlier diagnosis compared to non-functional NETs which often present fairly late with distant metastases or mass effect [4,6,7,8,9]. Most small intestinal (SI)-NETs produce serotonin and other vasoactive molecules which may cause carcinoid syndrome in the presence of liver metastases [6,10,11]. The most frequent symptoms in the carcinoid syndrome are flushing and diarrhea [11].

The CgA protein belongs to the Granin family. It is a precursor protein of 439 amino acids [12]. CgA immunoreactivity is found abundantly in neuroendocrine cells and increased plasma concentrations of CgA have been associated with most NETs [5,13,14]. However, a wide range of non-NET conditions are associated with elevated plasma CgA concentrations such as chronic atrophic gastritis, renal and hepatic dysfunction, cardiovascular disease, and rheumatologic disease. In addition, proton pump inhibitor (PPI) treatment is a common cause of CgA elevation [5,15]. On the other hand, not all NETs give rise to elevated plasma CgA. Important factors determining CgA concentrations are NET type, secretory activity and extent of disease [5,16,17]. Furthermore, diagnostic sensitivity and specificity depend on the assay type, analytic specificity and sensitivity of the antiserum employed [14], as does the fact that the CgA protein is heavily processed to smaller fragments [14,18,19,20].

In the ENETS (European Neuroendocrine Tumor Society) guidelines [21], measurement of CgA is recommended during follow-up of patients diagnosed with NET since plasma concentrations are related to tumor burden [14,18] and an increase in plasma concentration of CgA may be a predictor of disease progression [22,23]. Despite the diagnostic pitfalls, some guidelines [24,25,26] also recommend the use of plasma CgA in diagnosis of NETs, although no clinical data directly support this strategy. Therefore, we investigated the positive predictive value (PPV) and diagnostic utility of plasma CgA in patients referred to our NET Centre of Excellence for a thorough NET workup.

## 2. Methods

### 2.1. Patients

Patients were recruited from ENETS Centre of Excellence, Rigshospitalet, Copenhagen University Hospital. The NET Centre receives patients from eastern Denmark, which has roughly 2.7 million inhabitants. Patients referred from 1 January 2015 to 31 December 2019 with clinically suspected or histologically verified NET were included if plasma CgA measurements had been performed prior to referral and histological diagnosis. Exclusion criteria were: pre-referral plasma CgA concentration measured using another assay than our in-house CgA assay [18], pre-referral CgA measurement performed after histological verification of NET, and incomplete NET workup at the time of data collection. 

We collected data on age, gender, medical history, medication, renal function, pre- and post-referral CgA concentrations and outcome of examinations including imaging and pathology reports from patients’ files. 

The patients referred with a NET histologically verified prior to referral all had a Ki-67 < 20% corresponding to G1 or G2 NET. G3 NET and NEC patients [2] were referred to the Department of Oncology and therefore were not among the included patients. Patients with suspected NET of the pituitary, thyroid gland (medullary thyroid cancer), parathyroid glands and adrenals (paragangliomas and pheochromocytomas) were referred to our endocrine unit and were not included in the study.

The study was approved by the local data protection agency at Rigshospitalet (no. P-2019-225) and by the Danish Patient Safety Authority (no. 3-3013-3093/1). Due to the retrospective design informed consent was not required.

### 2.2. CgA_340-348_ Radioimmunassay

All measurements of plasma CgA concentration pre- and post-referral were performed using an in-house CgA_340-348_ radioimmunoassay (RIA) (CgA_340-348_). Upper reference limit (URL) is 130 pmol/L, based on a study population of 88 apparently healthy individuals of both sexes, median age 41 (interquartile range (IQR) 30–51) years [14]. In this assay, the antiserum employed binds an epitope that is located on sequence 340–348 [20]. Inter-assay coefficient of variation (CV) of replicate samples was estimated to 11% in a range from 7.5 to 100 pmol/L [18].

### 2.3. Processing-Independent Analysis of CgA

**Figure 1:**
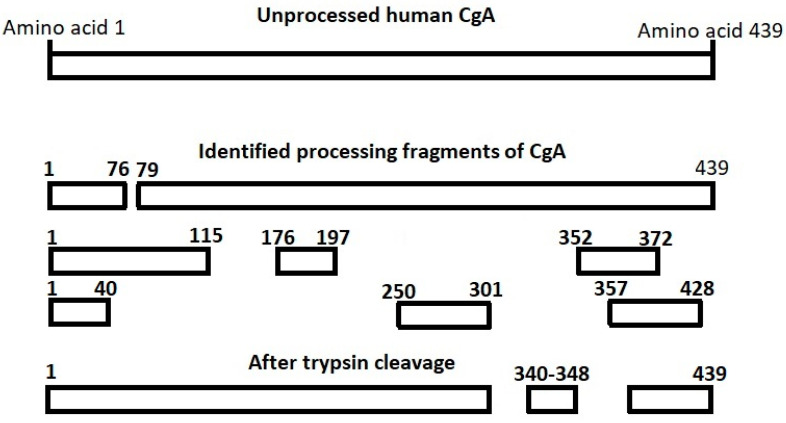
Identified processing products of chromogranin A (CgA). The fragments have been suggested to be bioactive with different primarily inhibitory effects. The CgA_340-348_ sequence does not undergo post-translational processing. CgA_340-348_ is located between tryptic cleavage sites and one CgA_340-348_ fragment will be released per CgA molecule when a sample is pre-incubated with trypsin, thus providing an accurate measure of the total CgA concentration of the sample.

The post-translational processing of the CgA protein is tissue-specific and varies individually. Therefore, measurement of a single CgA fragment in the post-translational processing cascade may lead to varying results among different patients [19]. To cope with such variability, processing-independent analysis has been developed [27] and also applied to CgA [14,18,19,27]. This method utilizes endoproteolytic cleavage of CgA by preincubating the sample with trypsin (hereby exposing the epitope at CgA_340-348_, which does not undergo post-translational processing) and subsequent quantification of CgA with the CgA_340-348_ RIA to quantify the entire mRNA translation product regardless of post-translational processing [14,18]. A diagram of CgA processing and fragments is shown in Figure 1. None of the CgA fragments have better diagnostic accuracy than processing-independent analysis (PIA) of CgA [18].

URL is 1100 pmol/L. This value was based on the same population as the CgA_340-348_ RIA [14]. From 2017 to 2019, CgA PIA was used in selected cases with unexplained continuously elevated CgA. If CgA PIA was within normal range, CgA_340-348_ was considered falsely elevated due to inter-individual variability of CgA expression and post-translational processing. Readers further interested in processing-independent analysis should study references 14, 18 and 19 [14,18,19].

### 2.4. Definitions

All NETs were diagnosed by histological examination of the primary tumor or metastases. If CgA was above the URL and NET was subsequently diagnosed, CgA was defined as true positive. If the CgA concentrations were within normal range and NET was diagnosed, CgA was defined as false negative. Correspondingly, if CgA was above the URL and no NET was diagnosed, CgA was defined as false positive and if CgA was within normal range and no NET was diagnosed, CgA was defined as true negative.

Subcategories of CgA elevation were defined: marginally elevated (1–2 × URL), moderately elevated (2–4 × URL), considerably elevated (4–8 × URL) and severely elevated (>8 × URL). The intervals were defined before the initiation of data analyses.

Renal dysfunction was defined as estimated glomerular filtration rate (eGFR) < 60 mL/min/1.73 m^2^. This cut-off was based on a study showing that non-NET patients with glomerular filtration rate (GFR) < 60 mL/min/1.73 m^2^ had significantly increased levels of CgA [28].

Symptoms leading to measurement of CgA concentrations in plasma were categorized into the following groups: Gastro-intestinal (GI) symptoms (diarrhea, constipation, abdominal pain, dyspepsia, nausea, vomiting), flushing, attack-like phenomena (palpitations, hypertension, dyspnea, symptoms of hypoglycemia or excessive sweating) and unspecific symptoms (night sweat, weight loss, fever, tiredness/fatigue). Other reasons for CgA measurement were examinations suggestive of NET (imaging or endoscopy) and coincidental.

### 2.5. Statistics

Statistical analysis and data collection were performed in IBM SPSS statistics 26. Positive predictive value (PPV) of pre-referral CgA was calculated as: patients diagnosed with NET who had elevated CgA concentrations in plasma divided by all patients with elevated CgA concentrations in plasma.

Diagnostic sensitivity was calculated as the number of patients diagnosed with NET who had elevated CgA concentrations divided by the total number of patients diagnosed with NET. 

The relationship between risk of NET and CgA plasma concentrations was assessed by a binary logistic regression analysis. Results are presented as odds ratio (OR) per 100 pmol/L increase in CgA plasma concentration.

When comparing CgA concentrations in patients before and after discontinuation of PPI, non-parametric statistical analysis (Wilcoxon signed rank test) was performed, since the data distribution was not Gaussian (even after logarithmic transformation (Log10)). Reported *p*-values are two-tailed and *p* < 0.05 is considered significant.

## 3. Results

### 3.1. Baseline Characteristics

**Figure 2:**
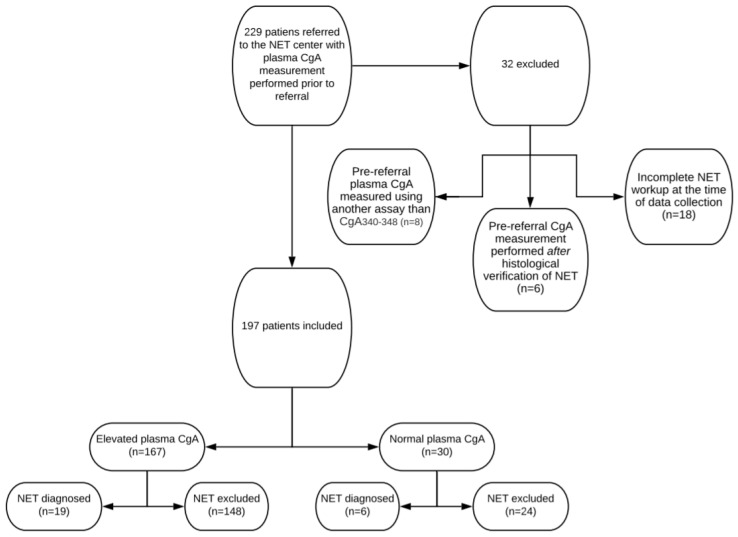
Flowchart of included and excluded patients, pre-referral CgA plasma concentration levels and total number of neuroendocrine tumors diagnosed.

Pre-referral CgA measurements were performed in 229 patients. Of these, 32 were excluded because pre-referral plasma CgA concentrations were measured using another assay than CgA_340-348_ (*n* = 8), pre-referral CgA measurements were performed after histological verification of NET (*n* = 6) or NET workup was incomplete at the time of data collection (*n* = 18). Thus, 197 patients were included in the final analysis (shown in Figure 2).

Baseline characteristics are shown in Table 1. Median age was 60 (range 20 to 88) years. Sixty-seven percent were female. Seven patients were referred to the NET Centre with histologically verified NET and 190 were referred with suspected NET for further workup. Plasma CgA concentrations prior to referral were elevated in 167 cases (85%) and normal in 30 cases (15%). Sixty-one (31%) were treated with PPI during the pre-referral measurement of CgA plasma concentrations. The indication for CgA measurement was based on symptoms in 154 cases (78%) and an examination suggestive of NET in 36 cases (18%) (imaging *n* = 28, endoscopy *n* = 8). In seven cases (3%), measurement of CgA was coincidental.

Of the seven patients referred with histologically verified NET, five had NET of the small intestine and two had NET of the pancreas.

Diagnostic imaging was performed less than 6 months prior to referral in 123 patients (60%) with a total of 145 imaging examinations. In 49 patients (24%) endoscopic examination was performed less than 6 months prior to referral with a total of 74 endoscopic examinations (shown in Table 2).

### 3.2. Examinations Post-Referral

In total 184 diagnostic examinations were performed post-referral, including 146 ^64^Cu-Dotatate or ^68^Ga-Dotatoc positron emission tomography (PET)/Computed tomography (CT) [29] (shown in Table 2). In 42 cases, no examination was performed post-referral for the following reasons: spontaneous normalization of plasma CgA concentrations post-referral (*n* = 20), normalization of CgA after discontinuation of PPI (*n* = 11), sufficient imaging performed pre-referral (*n* = 6), CgA PIA within the normal range (*n* = 5).

### 3.3. Positive Predictive Value Of Elevated CgA Prior to Referral

Twenty-five patients were diagnosed with NET either prior to referral but after measurement of CgA (*n* = 7) or after workup in the NET Centre (*n* = 18). The primary location was the small intestine (*n* = 16), pancreas (*n* = 6), adrenals (*n* = 2) and stomach (*n* = 1). NET cases are shown in Table 3. In patients with verified NET, CgA was within normal range (false negative) in six cases (24%) and above URL (true positive) in 19 cases, corresponding to a sensitivity of 76%. The sensitivity of an elevated CgA concentration was 50% in cases with localized disease, 80% in cases with local lymph node metastases and 100% in cases with distant metastases (shown in Table 3).

In 8 of 19 true positive cases, an elevated CgA contributed substantially to the NET suspicion. In the remaining 11 true positive cases, a suspected NET tumor was visualized by imaging (*n* = 10) or capsule endoscopy (*n* = 1) prior to CgA measurement.

The CgA concentrations were elevated without evidence of NET after workup in the NET Centre (i.e., false positive) in 148/197 cases (75%). NET was diagnosed in 19/167 cases with elevated CgA plasma concentration at referral. Thus, the overall positive predictive value (PPV) of CgA was 11% and increased to 15% if patients treated with PPI were excluded (16/109). PPV increased to 67% when the CgA plasma concentration was severely elevated, and patients treated with PPI were excluded. PPV of plasma CgA in patients treated with PPI was 5%. None of the six patients with severely elevated plasma CgA concentration during PPI treatment were diagnosed with NET (PPVs are shown in Table 4).

The plasma concentration of CgA was significantly associated with the risk of finding a NET; the OR was 1.12 per 100 pmol/L increase in plasma CgA concentration (*p* < 0.005). When excluding PPI-treated patients, the OR was 1.18 (*p* < 0.001). In patients treated with PPI, no significant association between CgA concentration and risk of NET was found—OR 0.96 (*p* = 0.74)

Table 5 shows how frequently a NET was diagnosed per indication for CgA measurement. When the indication for CgA measurement was an examination (i.e., imaging or endoscopy) suggestive of NET, 44% were diagnosed with NET. When CgA was measured because of GI symptoms 3% were diagnosed with NET.

### 3.4. Causes of Elevated CgA Apart From NET

In 39 cases with falsely elevated CgA, spontaneous normalization was observed between CgA measurements pre- and post-referral and in 19 cases comorbidity was identified as a possible cause of increased levels of CgA (renal dysfunction *n* = 10; heart disease *n* = 4; chronic atrophic gastritis *n* = 1, multimorbidity *n* = 4).

**Figure 3:**
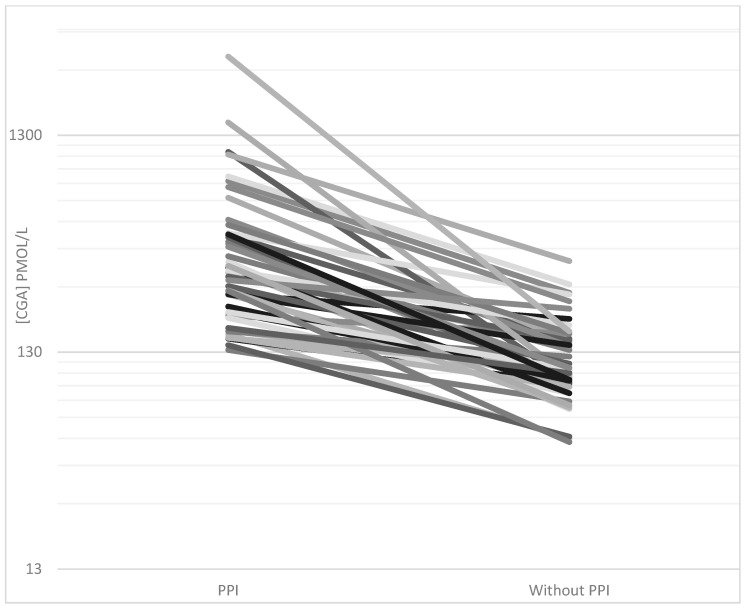
CgA plasma concentrations (pmol/L) in all patients with discontinued proton pump inhibitor (PPI) treatment. Plasma concentrations were measured using the CgA_340-348_ radioimmunoassay during PPI treatment (pre-referral) and after discontinuation of PPI for at least one week (post-referral).

CgA concentrations were elevated in 58/61 patients treated with PPI during pre-referral CgA measurements. When PPI was discontinued for at least 1 week, normalization of CgA was achieved in 28/46 cases. The average CgA concentration decreased from 445 (IQR 197–456) to 128 pmol/L (90–152) after discontinuation (*p* < 0.0001) (shown in Figure 3).

Processing-independent analysis of CgA (CgA PIA) was performed in 47 cases with unexplained continuously elevated CgA concentrations. In 28 patients, the CgA PIA was within normal range while simultaneously CgA_340-348_ was still elevated. In the remaining 19 cases, simultaneous plasma CgA_340-348_ and CgA PIA were both elevated (*n* = 17) or within normal range (*n* = 2). CgA PIA within normal range was used to disprove NET suspicion in five cases without performing other examinations. The remaining 42 patients had a ^64^Cu-Dotatate or ^68^Ga-Dotatoc PET/CT performed with no sign of NET.

## 4. Discussion

The main results from the present study were that only a small fraction—around 11%—of patients with elevated plasma CgA concentrations ended up with a histologically verified NET diagnosis after workup at a specialized tertiary center for diagnosing and treating G1 and G2 NETs. PPI treatment and differences in post-translational processing of CgA were identified as the most frequent causes of falsely elevated CgA concentrations. In accordance with previous studies, we found that normal CgA does not exclude NET, since 6 of 25 diagnosed patients did not have elevated plasma CgA [5,16].

In recent years, we have observed an increase in patients referred for NET workup with elevated concentrations of CgA. NET was only found in 19 out of 167 patients with elevated CgA. Furthermore, elevated CgA only contributed substantially to the NET diagnosis in eight of these cases since measurement of CgA in the remaining 11 cases was performed after imaging or endoscopy highly suggestive of NET. All patients with metastatic disease had elevated CgA concentrations whereas half of patients with localized disease had elevated CgA, strengthening the idea that CgA is more sensitive in disseminated disease compared to localized disease [16,17,18,30,31]. PPI is a well-known cause of falsely elevated CgA [5,13,15,32], but even after excluding the substantial number of patients treated with PPI, the overall PPV of elevated CgA was only 15%. The risk of NET was significantly associated with an increase in CgA plasma concentration and when plasma CgA was severely elevated, and PPI-treated patients were excluded, the PPV rose to 67%. There was no association between an increase in CgA concentration and NET risk when patients were treated with PPI. Therefore, it is questionable whether an elevated CgA concentration during PPI treatment should be assigned any diagnostic value.

We identified several causes of falsely elevated CgA concentrations. Around one-third of all cases with elevated CgA were treated with PPI and normalization of CgA was achieved after pausing PPI in around half of these cases. Furthermore, it is likely that a longer pause of PPI would have resulted in the normalization of CgA in more cases since prolonged treatment with PPI causes enterochromaffin-like (ECL)-cell hyperplasia [33,34]. In patients who cannot discontinue PPI, H2 receptor antagonist (H2RA) replacement may be tried during one or two weeks of PPI discontinuation [35] as H2RA affects gastric acidity, gastrin and CgA levels to a lesser extent than PPI [36]. In 39 patients, CgA was spontaneously normalized between CgA measurements pre- and post-referral, probably reflecting biological variability. Two studies have been conducted regarding biological variation of CgA both supporting that within-subject variability could have caused marginally elevated CgA, which would later normalize spontaneously [37,38]. In 19 cases, comorbidity was identified as a possible cause of increased levels.

As a novel approach, we measured CgA PIA in the patients with continuously unexplained elevated CgA concentrations in plasma. CgA PIA has demonstrated a high diagnostic accuracy (i.e., diagnostic sensitivity and specificity) compared to the CgA_340-348_ RIA and other CgA assays without trypsin cleavage prior to measurement [14,18]. In 60% of tested patients we found that CgA PIA was within normal range, while simultaneous CgA_340-348_ was still elevated, supporting a substantial improvement in diagnostic specificity. Thus, our data suggest that CgA PIA could be used to disprove NET suspicion based on elevated regular CgA, hereby avoiding unnecessary examinations. The CgA PIA is a cumbersome analysis to perform, and therefore it can only be requested by our NET Centre. Our data emphasize that new biomarkers of NENs are highly warranted. Circulating tumor mRNA has been investigated but more data are needed before these new technics can be recommend for clinical use [18,39].

A substantial delay in the diagnosis of NETs has been reported [7,40] highlighting the need for increased awareness of this rare type of tumor. Our data highlight the question of whether an increase in the use of diagnostic CgA measurement is the solution to this problem, since CgA measurements rarely contributed to the diagnosis and were most often falsely elevated. We therefore suggest that in case of symptoms suggestive of foregut NET (e.g., insulinoma or gastrinoma), the initial biochemical investigations should be restricted to the relevant specific hormone (e.g., insulin or gastrin). Regarding SI-NET, we observed that symptoms that could be associated with carcinoid syndrome rarely predicted NET. Therefore, we will argue against the routine use of plasma CgA measurement in patients with GI symptoms, flushing-like symptoms, or unspecific cancer symptoms. If a carcinoid tumor is suspected, we suggest performing a CT of the thorax and abdomen. SI-NETs rarely cause carcinoid syndrome before metastasizing to the liver, since the liver metabolizes bioactive molecules such as serotonin [10,11]. Therefore, a patient presenting with carcinoid syndrome most likely has disseminated disease with metastases visible on an abdominal CT. In alignment with this assumption, all SI-NETs were visualized when an abdominal CT prior to referral was performed (8 of 16 SI-NET patients—data not shown).

A strength of the present study is that it reflects the use of CgA in every day clinical practice in patients presenting with symptoms, which can be associated with NETs. Another strength is the extensive use of ^68^Ga-Dotatoc or ^64^Cu-Dotatate PET/CT, which was performed either prior to referral or predominantly in the NET Centre in more than 75% of patients. These imaging modalities are considered the most sensitive in diagnosing or excluding G1 and G2 NETs [29,41]. Furthermore, the same validated method and laboratory facilities were used for measurement of CgA in all analyses both pre- and post-referral. Our in-house CgA_340-348_ assay as well as the CgA PIA has recently been compared with eight commercially available kits in 130 well-characterized patients with SI-NETs. In terms of diagnostic accuracy, the in-house CgA_340-348_ RIA was superior to all commercial assays and in accordance with the results from the present study—the highest diagnostic accuracy was obtained with CgA PIA [18].

One limitation of this study is that we probably do not see the majority of true negative and maybe also false negative CgA measurements since a plasma CgA concentration within normal range presumably rarely leads to referral. This could lead to an overestimation of diagnostic sensitivity and an underestimation of diagnostic specificity. The negative predictive value and specificity of plasma CgA are undoubtfully much higher than what could be estimated based on our data and therefore these calculations have not been carried out.

In conclusion, our data do not support the use of CgA measurement as a screening tool in patients suspected of NET since the PPV was unacceptably low causing unnecessary examinations, delay in relevant examinations and worry for the patient. If a functional NET is suspected, we suggest measurement of relevant specific hormones and conducting relevant imaging. We observed a significant increase in the risk of finding a NET with increasing plasma concentration of CgA, suggesting that considering the degree of CgA elevation is important when evaluating NET risk. PPI should always be paused for at least one week before measurement of CgA since little diagnostic value can be assigned to even highly elevated CgA plasma concentrations during PPI treatment. CgA PIA was often within normal range while CgA_340-348_ RIA was falsely elevated, suggesting that this method could be a way of disproving NET suspicion when it is based primarily on elevated regular CgA plasma concentrations.

## Figures and Tables

**Table 1:** Baseline characteristics.

		Total	Normal CgA	[CgA] 1–2 × URL	[CgA] 2–4 × URL	[CgA] 4–8 × URL	[CgA]>8 × URL
**Patients included, *n* (%)**		197	30 (15%)	91 (46%)	41 (20%)	20 (10%)	15 (8%).
**Female, *n* (%)**		132 (67%)	17	63	29	12	9
**Age, years, median (IQR)**		60 (49–70)	59 (45–70)	56 (42–69)	63 (56–69)	61 (50–69)	71 (64–75)
**PPI-treated, *n* (%)**		61 (31%)	3	23	22	7	6
**NET at referral, *n***		7	1	2	0	2	2
**Referred for NET workup, *n***		190	29	89	41	18	13
**Main Indication for CgA measurement**	**GI symptoms, *n***	65 (33%)	4 (13%)	33 (36%)	18 (44%)	5 (25%)	5 (33%)
	**Attack-Like Phenomena, *n***	41 (20%)	5 (17%)	22 (24%)	8 (7%)	3 (15%)	2 (13%)
	**Examination suggestive of NET, *n***	36 (18%)	17 (57%)	10 (11%)	2 (5%)	4 (20%)	4 (27%)
	**Flushing, *n***	34 (17%)	4 (13%)	17 (19%)	7 (17%)	3 (15%)	3 (20%)
	**Unspecific symptoms, *n***	14 (7%)	0	8 (9%)	3 (7%)	2 (10%)	1 (7%)
	**Coincidental, *n***	7 (4%)	0	1 (1%)	3 (7%)	3 (15%)	0

Total number of included patients, age, number of proton pump inhibitor (PPI)-treated patients, number of patients with neuroendocrine tumor (NET) at referral and indications for CgA measurement grouped according to CgA elevation subcategories.

**Table 2:** Imaging and endoscopic examinations pre-and post-referral.

Pre- and Post-Referral Imaging								
	**CT Thorax and Abdomen**	**CT Abdomen**	**^18^F-FDG-PET/CT**	**MRI Abdomen**	**Transabdominal Ultrasound**	**^64^CuDotatate or ^68^GaDotatoc PET/CT**	**CT Thorax**	**^131^I-Meta-Iodbenzylguanidine (MIBG) Scintigraphy**
Pre-referral	54	31	27	10	9	6	3	0
Post-referral	1	4	8	10	1	146	0	1
Pre-and post-referral endoscopic examinations								
		**Colonoscopy**		**Gastroscopy**		**Capsule Endoscopy**		**Endoscopic Ultrasound**
Pre-referral		33		27		12		1
Post-referral		0		6		0		0

**Table 3:** NET location and extent of disease. Total number of patients and number of patients with CgA levels within normal range are given.

	Total	Localized	Lymph Node Metastases	Distant Metastases
**Total (normal CgA)**	25 (6)	10 (5)	5 (1)	10 (0)
**Small intestine (normal CgA)**	16 (2)	2 (1)	4 (1)	10 (0)
**Pancreas (normal CgA)**	6 (4)	5 (4)	1 (0)	0
**Adrenals (normal CgA)**	2 (0)	2 (0)	0	0
**Stomach (normal CgA)**	1 (0)	1 (0)	0	0

**Table 4:** Positive predictive value of CgA depending on PPI status and level of CgA elevation.

	Total	1–2 × URL	2–4 × URL	4–8 × URL	>8 × URL
All elevated CgA plasma concentrations (true elevated) and PPV, %	167 (19) 11%	91 (5) 5%	41 (2) 5%	20 (6) 30%	15 (6) 40%
Elevated CgA without PPI (true elevated) and PPV, %	109 (16) 15%	68 (4) 6%	19 (2) 11%	13 (4) 31%	9 (6) 67%
Elevated CgA with PPI (true elevated) and PPV, %	61 (3) 5%	23 (1) 4%	22 (0) 0%	7 (2) 29%	6 (0) 0%

**Table 5:** Indications for CgA measurements, frequency of NET diagnosis and NET type.

	Indication for CgA, *n*	NET Diagnosed, *n* (%)	NET Type, Location, Extent of Disease and CgA Level
**GI symptoms**	65	2 (3%)	Small intestine NET, distant metastases, elevated CgA (*n* = 1)
			ECL’oma, stomach, localized, elevated CgA (*n* = 1)
**Attack-like phenomena**	41	3 (7%)	Pheochromocytoma, localized, elevated CgA (*n* = 1)
			Insulinoma, localized, normal CgA (*n* = 1)
			Insulinoma, localized, elevated CgA (*n* = 1)
**Examination suggestive of NET**	36	16 (44%)	Small intestine NET, localized, normal CgA (*n* = 1)
			Small intestine NET, localized, elevated CgA (*n* = 1)
			Small intestine NET, lymph node metastases, normal CgA (*n* = 1)
			Small intestine NET, lymph node metastases, elevated CgA (*n* = 3)
			Small intestine NET, distant metastases, elevated CgA (*n* = 6)
			Pancreas NET, localized, normal CgA (*n* = 3)
			Pancreas NET, lymph node metastases, elevated CgA (*n* = 1)
**Flushing**	34	4 (12%)	Pheochromocytoma, localized, elevated CgA (*n* = 1)
			Small intestine NET, distant metastases, elevated CgA (*n* = 3)
**Unspecific symptoms**	14	0	
**Coincidental**	7	0	
